# An Algorithm for Network-Based Gene Prioritization That Encodes Knowledge Both in Nodes and in Links

**DOI:** 10.1371/journal.pone.0079564

**Published:** 2013-11-19

**Authors:** Chad Kimmel, Shyam Visweswaran

**Affiliations:** Department of Biomedical Informatics, University of Pittsburgh, Pittsburgh, Pennsylvania, United States of America; Tel Aviv University, Israel

## Abstract

**Background:**

Candidate gene prioritization aims to identify promising new genes associated with a disease or a biological process from a larger set of candidate genes. In recent years, network-based methods – which utilize a knowledge network derived from biological knowledge – have been utilized for gene prioritization. Biological knowledge can be encoded either through the network's links or nodes. Current network-based methods can only encode knowledge through links. This paper describes a new network-based method that can encode knowledge in links as well as in nodes.

**Results:**

We developed a new network inference algorithm called the Knowledge Network Gene Prioritization (KNGP) algorithm which can incorporate both link and node knowledge. The performance of the KNGP algorithm was evaluated on both synthetic networks and on networks incorporating biological knowledge. The results showed that the combination of link knowledge and node knowledge provided a significant benefit across 19 experimental diseases over using link knowledge alone or node knowledge alone.

**Conclusions:**

The KNGP algorithm provides an advance over current network-based algorithms, because the algorithm can encode both link and node knowledge. We hope the algorithm will aid researchers with gene prioritization.

## Introduction

Understanding the genetic and biological mechanisms of diseases is an ongoing challenge. Common diseases such as rheumatoid arthritis and breast cancer that occur relatively frequently in the population are likely to have complex and multifactorial underlying mechanisms. Moreover, common diseases likely arise from both genetic and environmental factors as well as from interactions among such factors. In recent years, several high-throughput techniques that survey a large number of genes have been developed for elucidating the genetic factors of common diseases. Such techniques include gene expression profiling, genotyping of single nucleotide polymorphisms, and whole genome sequencing to name just a few. One challenge with such techniques is that they typically produce hundreds of candidate genes associated with the disease of interest. To address this challenge, computational approaches have been developed for prioritizing candidate genes to reduce the number of promising genes that need to be examined in detail by the biomedical researcher.

### Candidate gene prioritization

Candidate gene prioritization is the process of identifying and ranking new genes as potential candidates of being associated with a disease or phenotype. Most candidate gene prioritization methods rely on a set of genes that are already known to be associated with the disease to rank the other genes. Genes that rank higher are more likely to be associated with the disease and more worthy of further biological investigation compared to those genes that rank lower. Developing excellent methods for candidate gene prioritization is important, because such methods can save biomedical researchers a significant amount of time, effort and resources by allowing them to focus on a relatively small set of promising genes to be studied in depth. Thus, candidate gene prioritization has enormous potential for accelerating progress in translational bioinformatics and in the development of new therapies.

The gene prioritization methods described in the literature can be broadly classified into two groups: similarity-based and network-based methods. Similarity-based methods attempt to identify those candidate genes whose features are most similar to genes that are already known to be associated with a particular disease. Examples of such features include expression patterns [1], [2], sequence features [3] and functional annotations [4]. More recently, network-based approaches have been developed and applied to candidate gene prioritization. In the next section, we describe in greater detail network-based methods, since the algorithm that we describe and evaluate in this paper is an example of a network-based method.

### Network-based methods

In the network-based approach to gene prioritization [5], [6], [7], [8], [9], [10], [11], [12], biological knowledge about genes is represented as a network. A network consists of nodes and links between pairs of nodes where nodes represent entities and links represent a variety of pair-wise relations that can exist among the entities. For example, in a protein-protein interaction network (PPIN), nodes represent proteins, and the links represent pair-wise interactions among the proteins. In a co-expression network, nodes represent genes whose expression levels are measured in a microarray experiment, and the links may represent correlations between expression levels of pairs of genes. We term a network, such as a PPIN, that incorporates knowledge as a knowledge network.

In network-based gene prioritization, an inference algorithm is applied to the knowledge network to rank genes (or proteins) relative to a root set of genes; members of the root set are genes that are known to be associated with a disease of interest. The premise underlying this approach is that genes in the network that are in close proximity to genes in the root set are more likely to be associated with the disease than those that are further away. Proximity between genes in a network can be defined and computed using a variety of inference methods and include methods that have been developed for social- and Web-network analysis such as PageRank [13] and Hyperlink-Induced Topic Search (HITS) [14].

Several investigators have examined network-based methods for gene prioritization. One of the earliest application of network-based gene prioritization was to rank each protein in the Online Predicted Human Interaction Database (OPHID) according to the protein's association with Alzheimer's disease [7]. Any gene which directly interacted with a known gene on the PPIN was considered to be a candidate gene – this is known as a “nearest neighbor” based approach. Even such a simple gene prioritization approach was shown to be effective. For example, a beta-catenin was predicted to be associated with Alzheimer's disease which had not been previously implicated in the disease. Since then, more sophisticated network algorithms have been applied. Kohler et al. [9] applied random walk and diffusion kernel network algorithms and Chen et al. [5] applied Web and social network algorithms to PPINs to prioritize candidate genes. Madi et al. developed a novel measure of node importance and used it to investigate antigen dependency networks computed from matrices of antigen–antigen correlations [15]. Furthermore, Madi et al. developed methods for identifying network components and their most informative interactions and applied them to networks of autoantibody reactivities in healthy mothers and their newborn babies [16].

Investigators have also integrated multiple knowledge sources to improve network-based gene prioritization. Frank et al. [17] constructed a classifier to predict interactions from a number of different data sources and used the classifier's output in the network. Chen et al. [18] combined different data sources including protein-protein interactions, gene expression data, and pathway data and showed that networks that used multiple data sources performed better than networks that used a single data source. A recent review provides a comprehensive overview of algorithms and tools including network-based methods used in gene prioritization [19]. Another recent review describes the application of network theory for the analysis and understanding of multi-level complex systems and discusses challenges for network-based science [20].

**Figure 1:**
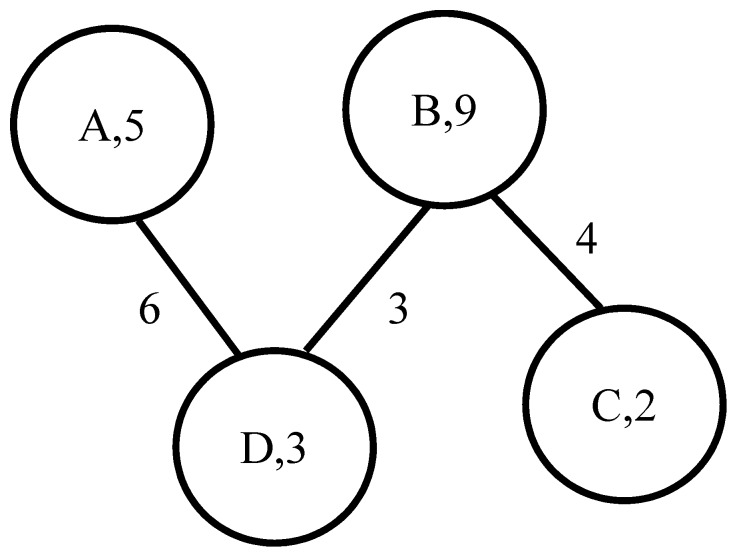
A small knowledge network.

One limitation of current network-based inference algorithms is that they utilize link weights but not node weights. However, knowledge about entities can also be represented as node weights in a knowledge network. We conjectured that an inference algorithm that utilized both link and node weights would perform better than an algorithm that only utilized link weights. Since there are no existing network-based inference algorithms that can utilize node knowledge, we developed a new network-based method called the Knowledge Network Gene Prioritization (KNGP) algorithm that utilizes link and node knowledge. As an illustrative example, consider the small knowledge network shown in Figure 1 where a link is annotated with a number that represents the link weight and a node is annotated with a number that represents the node weight. A typical network algorithm like PageRank when applied to this network to rank nodes A, B and C with respect to node D will rank A, B and C in that order because A's link to D has a higher link weight than B's link to D and C is only indirectly connected to D through B. A network algorithm that also considers the node weights may rank the nodes as B, A and C in that order because B's combination of node and link weights may be superior to A's combination of node and link weights.

### Knowledge Network Gene Prioritization (KNGP) algorithm

This section describes the KNGP algorithm in detail. KNGP creates a knowledge network from biological knowledge related to genes (or proteins). The biological knowledge is represented in two ways: 1) knowledge related to a gene is represented as a weight associated with the corresponding node (e.g., the number of gene ontology terms associated with a gene), and 2) knowledge related to a pair of genes is represented as a weight associated with the link that connects the corresponding nodes (e.g., whether the products of a pair of genes interact). For brevity, we call these node and link weights respectively. The algorithm outputs a ranking for the nodes relative to a set of genes already known to be associated with a disease of interest which is called the **root node set**. More specifically, the algorithm computes the posterior node importance for each gene in a set of genes called the **candidate node set**. The posterior node importance of a node is a measure of how likely the corresponding gene is to be associated with the disease of interest. The KNGP algorithm was motivated by the PageRank and the PageRank with Priors algorithms that are commonly used to rank nodes in a network.

PageRank and the PageRank with Priors algorithms were originally developed for networks with directed links, but have recently been applied to undirected networks. For application to an undirected network such as a PPIN, the network is converted into a directed network where an undirected link between two nodes is represented as two directed links. When PageRank is applied to an undirected network, the posterior node importance of a node is simply proportional to its degree (the number of neighboring nodes to which it is linked where the links are unweighted or the sum of the weights on the links where the links are weighted) [21], [22]. However, in PageRank with Priors or in personalized PageRank, the posterior node importance is not simply proportional to its degree and is computed using an iterative algorithm [21].

**Figure 2:**
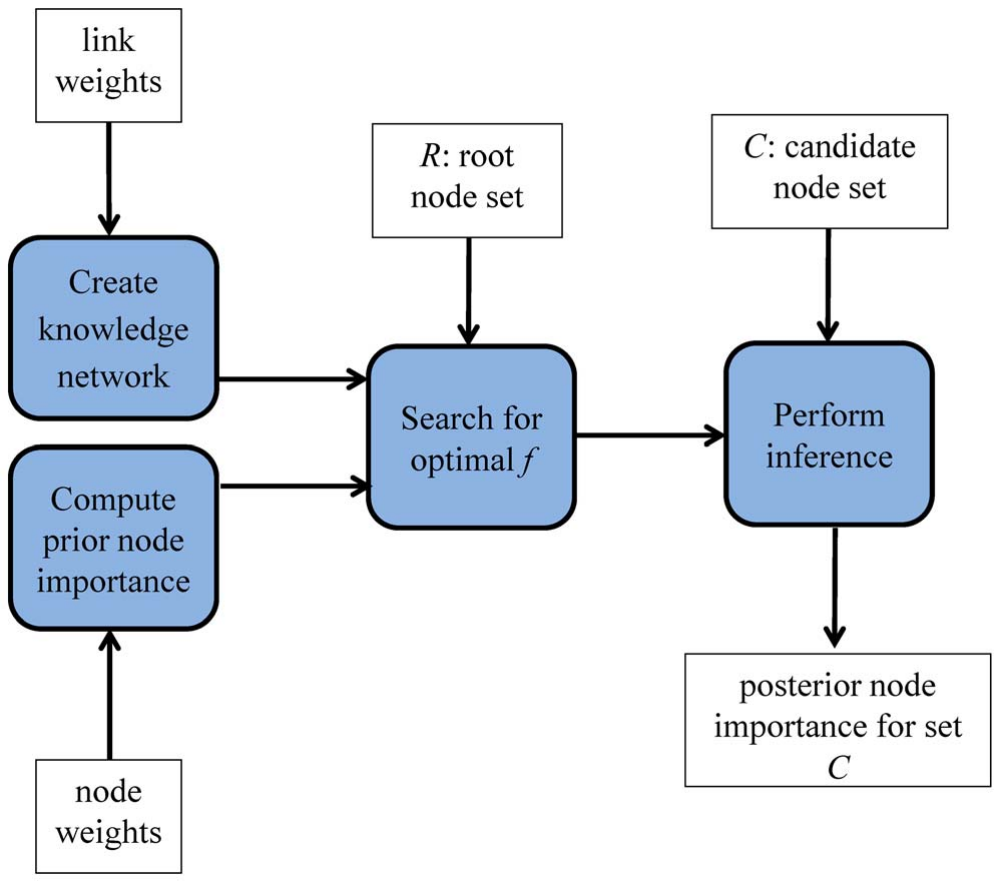
The components, inputs and output of KNGP algorithm.

**Figure 3:**
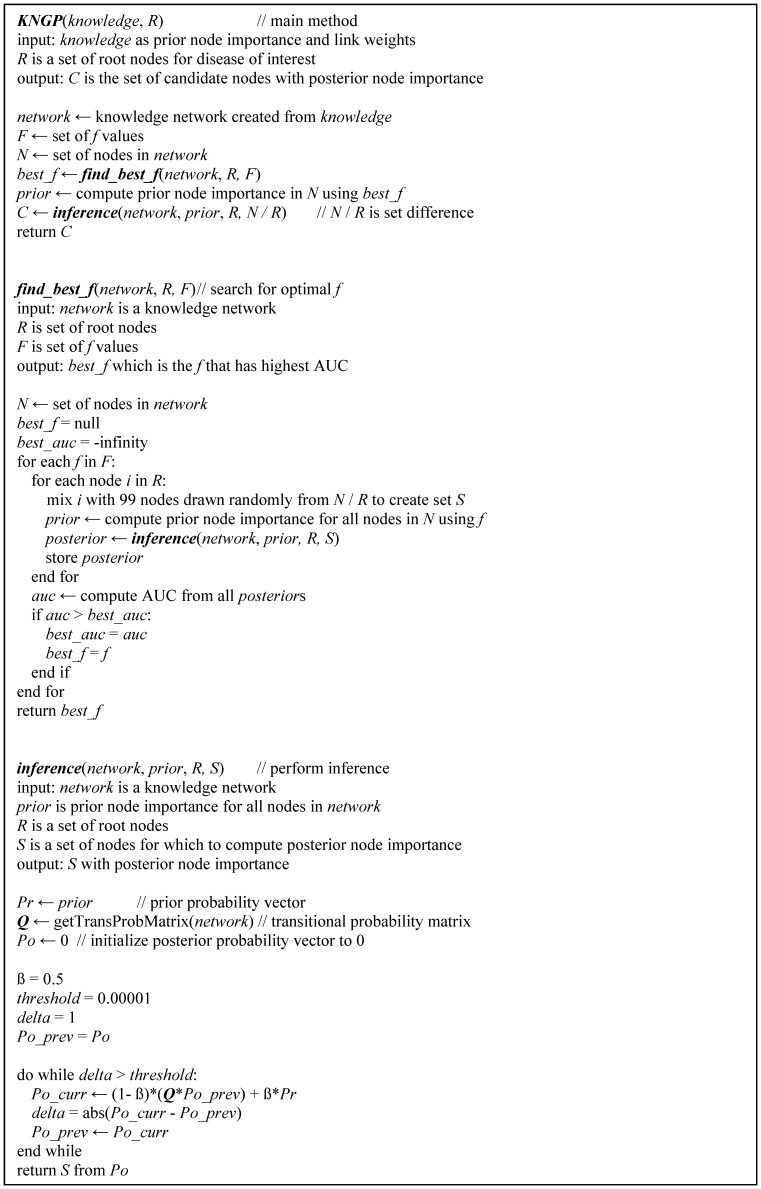
Pseudocode for the KNGP algorithm.

Figure 2 shows the components, inputs, and output of the KNGP algorithm and Figure 3 provides the pseudocode for the algorithm. The functions of the four components are to 1) create the knowledge network, 2) compute the prior node importance, 3) search for the optimal value of the parameter *f*, and 4) perform inference. The inputs include link weights, node weights, the set of root nodes *R* and the set of candidate nodes *C*. The output is the posterior node importance for each candidate node. We now describe the components of the KNGP algorithm in detail.

### Create the knowledge network

Two matrices are associated with the knowledge network: the link knowledge matrix and the transition probability matrix. **The link knowledge matrix** is a n*n matrix where *n* is the number of nodes in the knowledge network, and an entry in it represents the link weight between the nodes specified by the row number and column number. The **transition probability matrix** is a n*n matrix and is derived from the link knowledge matrix. An entry in this matrix denotes the transition probability of going to one node (represented by the row number) from another node (represented by the column number) in the network. The transition probability of going to node *v* from node *u* is given by:
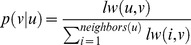
(1)where *lw*(*u*, *v*) is the link weight between node *u* and *v* obtained from the link knowledge matrix, and *neighbors*(*u*) is the set of neighboring nodes to which node *u* has a weighted link. If node *u* has no neighbors, then *p*(*v* | *u*) is set to 0, and by symmetry *p*(*u* | *v*) is also 0. This transition probability term encodes link knowledge.

### Compute the prior node importance

The prior node importance represents how likely – *a priori* – a given gene is associated with the disease of interest. The prior node importance is defined by two vectors: the node knowledge vector and the prior probability vector. The **node knowledge vector** is a *n* dimensional vector where *n* is the number of nodes in the knowledge network, and an entry in it represents the node weight associated with the corresponding node. The **prior probability vector** *Pr* is derived by normalizing the node knowledge vector. The prior probability *Pr_v_* of node *v* is defined as:
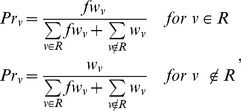
(2)where *R* is the set of root nodes, *w_v_* is the weight associated with node *v* that is obtained from the node knowledge vector, and *f* is a parameter that takes a value between 0 and positive infinity. The *f* scales the node weights for members of the root set compared to the non-root set. The next section describes how the optimal value of *f* is obtained. In summary, the prior probability term encodes both node knowledge and root node knowledge.

### Search for the optimal value of the parameter *f*

For a specific value of *f*, the KNGP algorithm performs inference to evaluate how highly the root nodes are ranked using leave-one-out cross-validation (described in the Methods section). Specifically, the performance associated with a value of *f* is measured using the area under the ROC curve (AUC).

The pseudocode for the search is given in the *find_best_f* procedure in Figure 3. The *find_best_f* procedure has three inputs: a network with link and node weights, *R* which is the set of root nodes, and *F* which is a set of *f* values defined by the user in the range 0 to positive infinity. As shown in the pseudocode, the outer loop iterates through *f* values in *F*, and the inner loop performs leave-one-out cross-validation to compute the AUC. The output of *find_best_f* procedure is the optimal value of *f* in *F* which is defined as the value that maximizes the AUC. The optimal *f* value depends on the relative distribution of the link and node weights between the root node and candidate node sets; hence, for a given knowledge network and disease of interest, the optimal *f* value can change.

### Perform inference

Given a network with a transitional probability matrix ***Q*** that encodes link knowledge, and a prior probability vector *Pr* that encodes both node knowledge with root node knowledge, inference on the network produces a posterior probability vector *Po* which is a *n* dimensional vector where *n* is the number of nodes in the network. KNGP's inference is based on a random walk model where a walker's probability of jumping from one node to another is proportional to the weight of the link that connects the two nodes. In addition, the probability of jumping from one node to another is modified by a “back probability” which determines how often the walker jumps back to the set of root nodes. The sequence of nodes visited during a random walk is represented by a Markov chain model. The relative number of visits to a node is obtained by computing the stationary probability of the Markov chain. The stationary probability distribution denotes the fraction of time that the walker spends at any one node during a random walk and is interpreted as the importance of the node relative to the other nodes in the network. The stationary probability distribution represents the posterior probability vector and is computed using the following iterative equation:
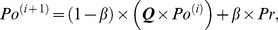
(3)where *Pr* is the prior probability vector, ***Q*** is the transitional probability matrix, *Po* is the posterior probability vector and ß is the back probability value inclusive between 0 and 1. *Po* is initialized to a vector of 0 s at the start of inference. At iteration *i*+1, *Po* is updated by multiplying *Po* at iteration *i* with the matrix ***Q***. The stationary distribution is reached when the difference in the elements of *Po* at (*i*+1) and *Po* at *i* falls below a small constant *delta*.

The posterior probability vector includes a probability for every node in the network. After the stationary posterior probability vector is obtained, the KNGP algorithm ranks the candidate nodes and outputs them along with their posterior probabilities. Often times, the candidate nodes will consist of all nodes in the network that are not in the root node set.

### PageRank with Priors algorithm

The network-based algorithm which is most similar to KNGP is PageRank with Priors (PRP). PRP first takes as input a network and a root node set. The algorithm then computes a relative importance score for each of the remaining nodes in the network. The PRP algorithm was originally applied to assign importance to webpages on the World Wide Web in relation to a specified set of webpages [23]. In PRP, the prior probability *Pr_v_* of node *v* is defined as:
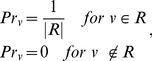
(4)where |*R*| is the number of nodes in the root set. It is important to note that (4) does not have a term to introduce node knowledge. Rather, only root node knowledge is incorporated into the prior probabilities. Similar to the KNGP algorithm, PRP uses equation 3 to perform inference. The main difference between the two algorithms lies in the prior probabilities.

Chen et al. [5] applied PRP to candidate gene prioritization and showed that network-based methods which previously had been used to study primarily social and web networks are also applicable to gene prioritization. As described earlier, PageRank with Priors is applied to an undirected network by converting the undirected link to two directed links.

## Methods

This section provides details of the datasets and the experimental setup to evaluate the KNGP algorithm.

### Synthetic networks

We created several synthetic networks with the goal of investigating how the node weights interacted with the link weights to influence the AUC at different *f* values in the KGNP algorithm.

The synthetic datasets were created as follows. Each dataset contained 1000 nodes of which nodes 1 to 100 are designated as root nodes and the remaining nodes are designated as candidate nodes (or non-root nodes). To assign node weights and link weights, the 1000 nodes were partitioned into the following 5 groups:

- Group 1 consisted of root nodes 1 through 50
- Group 2 consisted of root nodes 51 through 10
- Group 3 consisted of candidate nodes 101 through 150
- Group 4 consisted of candidate nodes 151 through 200
- Group 5 consisted of candidate nodes 201 through 1000

Four datasets were generated in the following manner:

- In dataset 1, each of the 1000 nodes was assigned a random node weight between 0 and 1. Thus, root nodes and candidate nodes had similar node weights. The links among the root nodes (i.e., node groups 1 and 2) were assigned a random weight between 0.5 and 1 and the links among the candidate nodes and among the root nodes and the candidate nodes were assigned a random weight between 0 and 0.5. Thus, links among root nodes had higher weights than other links.
- In dataset 2, the root nodes (i.e., groups 1 and 2) were assigned a random node weight between 0.5 and 1, and the candidate nodes (i.e., groups 3, 4 and 5) were assigned a random node weight between 0 and 0.5. Thus, root nodes had higher node weights than all of the candidate nodes. All links were assigned a random link weight between 0 and 1. Thus, links among root nodes, links among candidate nodes and links among root nodes and candidate nodes had similar weights.
- In dataset 3, the root nodes were assigned a random weight between 0.9 and 1.0, and the candidate nodes were assigned a random weight between 0.5 and 1.0. Thus, the root nodes, on average, had higher node weights than the candidate nodes, but some of the candidate nodes could have had greater node weights. The link weights between the root nodes were assigned a value between 0.55 and 1.0, and the link weights between the candidate nodes were assigned a value between 0.5 and 1.0. Thus, the links between the root nodes were, on average, higher than the link weights between the candidate nodes, but some of the candidate node link weights could have been higher.
- In dataset 4, the root nodes were assigned a random node weight between 0.95 and 1.0, and the candidate nodes were assigned a random node weight between 0 and 1.0. Thus, the root nodes, on average, had higher node weights than the candidate nodes, but some of the candidate nodes could have had greater node weights. The link weights between the root nodes were assigned a value between 0.1 and 1.0, and the link weights between the candidate nodes were assigned a value between 0 and 1.0. Thus, the links between the root nodes were, on average, higher than the link weights between the candidate nodes, but some of the candidate node link weights could have been higher.

The KNGP algorithm was run on each of the synthetic networks using the evaluation protocol (described in Methods section) for a range of *f* parameter values that included the following: 0, 1, 15, 100, 10,000 and 10^10^. At one extreme, *f* = 0, the prior probabilities of the root nodes became 0, and the prior probabilities of the candidate nodes were proportional to the node weights. At the other extreme, *f* = 10^10^, the prior probabilities of the root nodes approached infinity, and the prior probabilities of the candidate nodes approached 0 due to normalization. Table 1 shows the link weights that were used for each dataset. Table 2 and Table 3 show the link weights that were used for each individual group and between the groups respectively. In creating synthetic networks, we did not include complex topologies that may arise from grouping nodes into groups or modules. Such grouping may be useful in the analysis of PPINs where groups of proteins may represent metabolic pathways or functional modules.

**Table 1:** Specification of node weights for each group in the synthetic networks.

Dataset	Node Weights				
	Group 1	Group 2	Group 3	Group 4	Group 5
1	rand(0,1)	rand(0,1)	rand(0,1)	rand(0,1)	rand(0,1)
2	**rand(0.5,1)**	**rand(0.5,1)**	rand(0,0.5)	rand(0,0.5)	rand(0,0.5)
3	**rand(0.9,1)**	**rand(0.9,1)**	rand(0.5,1)	rand(0.5,1)	rand(0.5,1)
4	**rand(0.95,1)**	**rand(0.95,1)**	rand(0,1)	rand(0,1)	rand(0,1)

**Table 2:** Specification of link weights for each group in the synthetic networks.

Dataset	Link Weights				
	Group 1	Group 2	Group 3	Group 4	Group 5
1	**rand(0.5,1)**	**rand(0.5,1)**	rand(0,0.5)	rand(0,0.5)	rand(0,0.5)
2	rand(0,1)	rand(0,1)	rand(0,1)	rand(0,1)	rand(0,1)
3	**rand(0.55,1)**	**rand(0.55,1)**	rand(0.5,1)	rand(0.5,1)	rand(0.5,1)
4	**rand(0.1,1)**	**rand(0.1,1)**	rand(0,1)	rand(0,1)	rand(0,1)

**Table 3:** Specification of link weights between groups in the synthetic networks.

Dataset	Link Weights									
	Group 1–2	Group 1–3	Group 1–4	Group 1–5	Group 2–3	Group 2–4	Group 2–5	Group 3–4	Group 3–5	Group 4–5
1	**rand(0.50,1)**	rand(0,0.50)	rand(0,0.5)	rand(0,0.5)	rand(0,0.5)	rand(0,0.5)	rand(0,0.5)	rand(0,0.5)	rand(0,0.5)	rand(0,0.5)
2	rand(0,1)	rand(0,1)	rand(0,1)	rand(0,1)	rand(0,1)	rand(0,1)	rand(0,1)	rand(0,1)	rand(0,1)	rand(0,1)
3	**rand(0.55,1)**	rand(0.50,1)	rand(0.5,1)	rand(0.5,1)	rand(0.5,1)	rand(0.5,1)	rand(0.5,1)	rand(0.5,1)	rand(0.5,1)	rand(0.5,1)
4	**rand(0.10,1)**	**rand(0.10,1)**	rand(0,1)	rand(0,1)	rand(0,1)	rand(0,1)	rand(0,1)	rand(0,1)	rand(0,1)	rand(0,1)

### Biological networks

We created several networks from biological knowledge. One set of networks encoded link knowledge derived from protein-protein interactions and from the Gene Ontology (GO) annotations. Another network encoded node knowledge that was derived from the GO annotations. And a final network encoded both link knowledge and node knowledge. All the networks had the same set of links where the presence of a link indicated a protein-protein interaction. Our goal was to evaluate the additional benefit of encoding node knowledge for gene prioritization using the KGNP algorithm.

#### Protein-Protein Interaction+GO link weight networks

The nodes in these networks represented genes (proteins), and a link was present between two genes if there was a protein-protein interaction (PPI) between the corresponding proteins. We obtained PPIs from the Interologous Interaction Database (IID) [24], [25] and the human protein-protein interaction (HPPI) database [26], [27]. In total, the networks contained 126,668 interactions between 11,259 proteins. The weight for a link was obtained from the Gene Ontology (GO) and is described next.

The GO [28] is a set of controlled vocabularies which describes the functions of proteins within the cell. The GO is divided into three separate ontologies that describe molecular function, biological process, and cellular component. Given a specific GO ontology such as GO molecular function, we calculated the similarity between a pair of genes using the algorithm described in Wang et al. [29]. This algorithm measures the functional similarity of two genes based on the semantic similarities among the GO terms annotating these genes. It encodes a GO term's semantics into a numeric value by aggregating the semantic contributions of their ancestor terms in the GO graph and uses this numeric value to measure the semantic similarity of two GO terms.

We created three networks with link weights corresponding to the three GO ontologies that are labeled as the PPI+GOM (with weights derived from the GO molecular function ontology), the PPI+GOB (with weights derived from the GO biological process ontology) and PPI+GOC (with weights derived from the GO cellular component ontology).

#### GO node weight network

This network was obtained by augmenting the PPI network with node weights. The PPI network was constructed as described above for the link weight networks. A node's weight represented the number of GO terms associated with the corresponding gene. To obtain the node weight for a gene, we totaled the number of terms obtained from all three gene ontologies including the cellular, molecular and functional ontologies.

#### Combined link and node weight network

This network was obtained by combining the PPI+GOC link weight network with the GO node weight network. The link weights in this network were the same as those used in the PPI+GOC network, and the node weights were the same as those used in the GO network.

#### Root nodes

The root nodes consisted of genes known to be associated with the disease of interest. We selected 19 diseases (see Table 4) and obtained the genes known to be associated with each disease from the Genetic Association Database (GAD) [30]. The GAD contains both positive and negative gene-disease associations. A positive association asserts that the gene is associated with the disease of interest, and a negative association asserts that the gene is not associated with the disease of interest. We included a gene in the root set for a disease if the gene had two more positive associations than negative associations in the GAD. A list of the known proteins associated with each disease that we used in our experiments is given in Appendix S1.

**Table 4:** Number of genes known to associated with each of the 19 experimental diseases.

Disease	Number of genes
Rheumatoid Arthritis	24
Parkinson's Disease	21
Celiac Disease	16
Esophageal Cancer	8
Hepatitis C	8
Crohn's Disease	17
Breast Cancer	27
Asthma	29
Alzheimer's Disease	21
Ulcerative Colitis	24
Endometriosis	5
Lymphoma	7
Osteoarthritis	8
Epilepsy	6
Atherosclerosis	43
Pancreatitis	6
Cirrhosis	7
Myocardial Infarction	32
Tuberculosis	12

### Evaluation

We used a leave-one-out cross-validation scheme where each root node was “left-out” - in turn - from the root node set. The KNGP algorithm was then applied to the network to determine how highly the left-out root node was ranked. The higher the left-out root node was ranked; the better was the performance of the KNGP algorithm.

**Figure 4:**
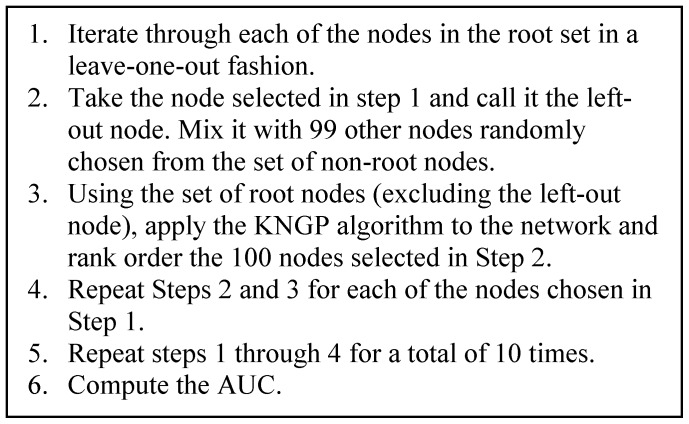
Evaluation protocol.

The leave-one-out evaluation protocol is shown in Figure 4. The protocol generates a total of m*10 (where *m* is the size of the root node set) rank ordered lists of 100 nodes each with a left-out root node that is embedded in 99 non-root nodes. A threshold rank (for example, the 5th rank) for such a list separates those nodes that are ranked above it from those that are ranked below it. For a given threshold rank, sensitivity is defined as the percentage of lists where the left-out node was ranked above the threshold and specificity as the percentage of lists where the left-out node was ranked below the threshold. Varying the threshold rank produced a series of sensitivity and specificity values from which a ROC curve was constructed, and the corresponding AUC was calculated.

We applied the KNGP algorithm to each of the synthetic networks and the biological networks using the evaluation protocol.

## Results

This section provides the results that we obtained for the synthetic and biological networks.

### Synthetic networks

As Table 5 shows, the optimal *f* value (i.e., the *f* value that achieved the highest AUC) depends on the degree to which the link and node weights are biased towards the root nodes versus the non-root nodes. In this context, the bias indicated how much greater the node or link weights were for the root nodes versus the non-root nodes. If the link weights were considerably more biased towards the root nodes than the non-root nodes – as in dataset 1 – than the highest AUC was obtained at the largest *f* value. Conversely, if the node weights were considerably more biased towards the root nodes than the non-root nodes – as in dataset 2 – than the highest AUC was obtained at the smallest *f* value. When the bias towards the root nodes was more balanced between the node weights and link weights – as in datasets 3 and 4 – than the highest AUC was obtained at a *f* value between the two extremes.

**Table 5:** AUCs for various values of *f* for the four synthetic datasets.

Dataset	*f* = 0	*f* = 1	*f* = 15	*f* = 100	*f* = 10,000	*f* = 10^10^
1	0.602	0.651	0.991	**1.000**	**1.000**	**1.000**
2	**1.000**	0.999	0.996	0.877	0.467	0.461
3	0.898	0.901	0.941	**0.977**	0.924	0.922
4	0.974	0.978	**0.991**	0.975	0.897	0.895

These results provide some intuition for the *f* parameter in the KGNP algorithm. The *f* parameter represents the tradeoff in importance between the link weights and the node weights in determining the ranking of the nodes. If the optimal *f* value is high, then it implies that the link weights dominate over the node weights in determining the ranking. Conversely, if the optimal *f* value is low, then it implies that the node weights dominate over the link weights in determining the ranking. These results imply that when the optimal *f* value occurs between the two extremes, both node and link weights are used to determine the ranking equally. Conversely, at the extremes, either the node weight or the links weights are used almost exclusively.

### Biological networks

Table 6 provides the AUCs values for each of 19 diseases obtained by applying KNGP to the three link weight networks. Of the three GO link weight networks, PPI-GOC performed the best and we used this network for creating the combined link and node weight network. Table 7 provides the AUCs values for each of 19 diseases obtained by applying KNGP to PPI-GOC link weight network, GO node weight network and a network that combines PPI-GOC link weights with GO node weights. The last row in the last column in Table 6 gives the p-values obtained from the two-tailed Wilcoxon paired-samples signed-rank test comparing the combined network with the link weight network and the node weight network. The combined network has significantly better performance at the 0.05 significance level than either the link weight network or the node weight network.

**Table 6:** AUCs for biological networks that contain weighted links.

Disease	PPI+GOM	PPI+GOB	PPI+GOC
Rheumatoid Arthritis	0.750	0.830	0.798
Parkinson's Disease	0.652	0.668	0.668
Celiac Disease	0.744	0.814	0.795
Esophageal Cancer	0.840	0.871	0.858
Hepatitis C	0.502	0.764	0.759
Crohn's Disease	0.850	0.862	0.846
Breast Cancer	0.866	0.872	0.865
Asthma	0.797	0.856	0.825
Alzheimer's Disease	0.807	0.843	0.828
Ulcerative Colitis	0.740	0.706	0.738
Endometriosis	0.747	0.953	0.944
Lymphoma	0.770	0.875	0.872
Osteoarthritis	0.840	0.778	0.837
Epilepsy	0.579	0.622	0.612
Atherosclerosis	0.880	0.840	0.827
Pancreatitis	0.852	0.715	0.865
Cirrhosis	0.525	0.689	0.683
Myocardial Infarction	0.884	0.892	0.880
Tuberculosis	0.800	0.887	0.876
**Average**	**0.757**	**0.807**	**0.809**

**Table 7:** AUCs for biological networks with link weights only, node weights only and combined link and node weights.

Disease	PPI+GOC link weight network	GO node weight network	PPI+GOC and GO combined network
Rheumatoid Arthritis	0.798	0.770	0.835
Parkinson's Disease	0.668	0.724	0.734
Celiac Disease	0.795	0.775	0.807
Esophageal Cancer	0.858	0.876	0.853
Hepatitis C	0.759	0.774	0.756
Crohn's Disease	0.846	0.808	0.847
Breast Cancer	0.865	0.855	0.867
Asthma	0.825	0.794	0.845
Alzheimer's Disease	0.828	0.868	0.863
Ulcerative Colitis	0.738	0.701	0.740
Endometriosis	0.944	0.758	0.986
Lymphoma	0.872	0.910	0.918
Osteoarthritis	0.837	0.803	0.858
Epilepsy	0.612	0.710	0.718
Atherosclerosis	0.827	0.885	0.896
Pancreatitis	0.865	0.755	0.878
Cirrhosis	0.683	0.579	0.666
Myocardial Infarction	0.880	0.885	0.907
Tuberculosis	0.876	0.833	0.943
**Average**	**0.809**	**0.793**	**0.838**
p-value			0.02/0.02

### Application to Asthma

Table 8 gives the top 5 ranked candidate genes for asthma that were obtained by applying the KNGP algorithm to the combined PPI-GOC and GO network. The two proteins – IL9R and IL12B – that are shown in bold font in Table 8 were ranked far lower by the other two networks. We obtained evidence from the literature that both these proteins have an association with asthma.

**Table 8:** Top five ranked candidate proteins for asthma.

**Q01113 (IL9R)**
Q13224 (GRIN2B)
P24394 (IL4R)
P29460 (IL12B)
P48357 (LEPR)

Kauppi et al. [31] genotyped several alleles from the IL9R gene and compared results between a large cohort of patients with asthma and healthy-control samples. The results were studied using linkage analysis, transmission disequilibrium, and homozygosity analyses. The authors showed that a IL9R allele – sDF2*10 – was more likely to be transmitted among patients with asthma and was found homozygotic among asthma patients more often than expected. Furthermore, a specific X chromosome haplotype was found to be more associated for patients with asthma. In order to test the hypothesis that the IL12B gene contains polymorphisms associated with asthma, Randolph et al. [32] performed a genotype analysis for polymorphisms in the IL12B gene between patients with asthma and their parents. In the results, the authors showed that one of the alleles of the IL12B gene was under-transmitted to children with asthma. Furthermore, the authors showed that a polymorphism of the IL2B gene may be significantly associated with asthma severity in whites.

Appendix S2 provides the top 10 ranked candidate proteins for each of the 19 experimental diseases obtained by applying the KNGP algorithm to the combined PPI+GOC and GO network.

## Discussion

Developing effective computational methods for candidate gene prioritization is an important problem in bioinformatics. In this paper, we presented and evaluated a new network-based method called the KNGP algorithm. The advantage of the KNGP algorithm is that it can encode node knowledge in addition to link knowledge into the network-based gene prioritization process and thus represents an advance over current network-based gene prioritization algorithms. On 19 diseases, we showed that the incorporation of link and node knowledge can add a significant benefit to the network-based gene prioritization process. We applied the new network-based method that we have introduced to PPINs; however, we anticipate that it is applicable to a range of other molecular and biological networks such as gene networks, metabolic networks and neural networks. Beyond biological networks, this algorithm will likely be useful in the analysis of Web and citation networks and other social and financial networks.

A main limitation of the current paper is that the KNGP algorithm searches over only a limited number of fixed values for the *f* parameter. We restricted the search to a few values to decrease the running time of the algorithm. A more advanced searching algorithm may lead to more optimal performance, but our experience indicated that the difference would not be too significant since the search space is highly convex.

In this paper, we explored only protein-protein interactions and GO annotations for link weights and GO annotations for node weights as the knowledge sources. Exploring alternative types of knowledge sources for the node and link weights may lead to better performance and is a possible extension for further research. Another extension is to combine the rankings from various networks derived from different knowledge sources. In the future, we plan on exploring these different research avenues.

## Conclusions

We presented a new network-based algorithm that is able to incorporate different types of biological knowledge in nodes and in links called the KNGP algorithm. Our results indicate that encoding both node and link knowledge can improve performance over using only link knowledge in network-based gene prioritization. We hope that researchers will find our new network-based approach useful for candidate gene prioritization and that future extensions will yield additional improvements.

## Supporting Information

Appendix S1 Uniprot identifiers of known proteins associated with each disease that was obtained from the Genetic Association Database.
(DOCX)

Appendix S2 Uniprot identifiers of the top 10 ranked candidate proteins for each of the 19 experimental diseases.
(DOCX)
