# Supplementary material for: An Algorithm for Network-Based Gene Prioritization That Encodes Knowledge Both in Nodes and in Links
Source: PLoS One. 2013 Nov 19;8(11):e79564. doi: 10.1371/journal.pone.0079564 (PMC3834271; doi:10.1371/journal.pone.0079564)
Supplement: Appendix S1 — Uniprot identifiers of known proteins associated with each disease that was obtained from the Genetic Association Database. (DOCX) [file pone.0079564.s001.docx]

**Appendix A**

Uniprot identifiers of known proteins associated with each disease that was obtained from the Genetic Association Database.

| **Rheumatoid Arthritis** | **Parkinson's Disease** | **Celiac Disease** | **Esophageal Cancer** | **Hepatitis C** | **Crohn’s Disease** |
| --- | --- | --- | --- | --- | --- |
| P20039  P21580  P08700  Q03519  Q96A65  Q9UNS1  P01909  P49279  P51681  Q15116  Q14116  P22301  P16410  O75015  P08637  P01920  P01584  Q7RTU3  P18510  P31939  P19438  Q96P31  P01579  P08254 | P29475  P27338  P04062  P15559  Q5S007  P00326  P07339  Q9H1E3  P10635  Q8IUH8  Q92731  P50406  P23560  P10636  P27169  Q9BXM7  P43354  P09488  O60260  P52824  P06307 | P29459  P01920  O95256  Q9HBE4  Q13478  Q01638  Q08116  Q04864  P01909  P16410  Q9Y6W8  P60568  P32246  P51677  Q2LD37  Q9UQQ2 | P04818  P24385  P42898  P15559  P05091  P34896  O14965  P04798 | Q8IZI9  P20591  Q8IZJ0  P16410  P10914  P01130  P30685  Q30201 | P20039  P17706  P08571  P35408  P54652  Q8TAU0  Q14116  Q9H015  O76082  P08183  P19438  P00738  P26927  P01375  Q5VWK5  P14174  Q676U5 |
| **Breast Cancer** | **Asthma** | **Alzheimer’s Disease** | **Ulcerative Colitis** | **Endometriosis** | **Lymphoma** |
| P20815  P05121  P08183  P03372  P04637  P05164  P27169  P50225  P09211  P04179  P06401  P38398  Q16678  P05093  P22455  P21802  P39060  P16035  P33241  Q13233  P05106  P04798  P29474  P11473  Q14790  P01579  P08253 | P01024  Q9GZX7  P60022  P21731  P05121  Q15746  P36222  P01375  P01920  P20930  Q9NQ38  Q9BZ11  P13500  P13501  P01909  Q14116  P35225  P04440  P05112  P10145  Q14765  P51677  P05106  P29475  P11684  Q9UIL8  P01011  P01579  P14780 | P02654  P21397  O96008  P01375  P05164  P78380  Q15165  Q12800  P10909  Q9BZA7  P01584  Q03014  Q9Y6A2  P02649  P01034  P30533  P28223  P30456  P06276  P49768  P04406 | P20039  P40879  P09622  P12318  P08571  P01903  Q9NZK7  Q8TAU0  Q9NPH9  P07942  O00206  Q14116  P22301  P20809  P08183  Q9H257  P26927  P01375  Q5VWK5  P14174  P01579  Q9NXI6  Q9UIR0  Q0VDK5 | P04440  P06401  P15692  P01909  P03372 | P04637  P22301  P16410  P24394  Q9UNQ0  P10415  P41182 |
| **Osteoarthritis** | **Epilepsy** | **Atherosclerosis** | **Pancreatitis** | **Cirrhosis** | **Myocardial Infarction** |
| P24394  Q9BXN1  P02458  P11473  P43026  P02452  P01583  Q9UEF7 | P01213  P23560  O95180  Q8N135  P35498  P18507 | P13498  P02656  P29474  P08571  P16284  Q07869  P02741  P49238  P45452  P04035  P11150  P11597  P34913  Q6Q788  P02647  P02649  P09601  P05231  P23946  P05362  P12821  Q9BQB6  O60603  P07204  P07203  Q15848  P30556  P04180  P01344  P35520  P16109  P78380  P27169  P35568  Q15165  P01303  P14780  P08253  P08254  Q9UEF7  P06858  Q8TE73  P01019 | P20039  P07477  O00206  P00995  P05091  P13569 | P05091  Q5SRN2  P01375  P01920  P05019  Q9UIR0  Q30201 | P40225  P42772  P29474  P15692  P02741  P02649  P04114  P07359  P16442  P05362  P42771  P51681  P08514  P07204  P03372  P41597  P00488  Q15848  P12821  P07996  P00748  P16284  P05019  P30533  P78380  P27169  Q15165  P08254  P11712  P05121  P06858  P01019 |
| **Tuberculosis** |  |  |  |  |  |
| P20039  Q99572  P11473  P42701  P01909  P10145  P49279  O60603  P22301  P29460  P01920  P01579 |  |  |  |  |  |
