# Supplementary material for: An Algorithm for Network-Based Gene Prioritization That Encodes Knowledge Both in Nodes and in Links
Source: PLoS One. 2013 Nov 19;8(11):e79564. doi: 10.1371/journal.pone.0079564 (PMC3834271; doi:10.1371/journal.pone.0079564)
Supplement: Appendix S2 — Uniprot identifiers of the top 10 ranked candidate proteins for each of the 19 experimental diseases. (DOCX) [file pone.0079564.s002.docx]

**Appendix B**

Uniprot identifiers of the top 10 ranked candidate proteins for each of the 19 experimental diseases obtained by applying the KNGP algorithm to the PPI+GOC and GO node weight network.

| **Rheumatoid Arthritis** | **Parkinson's Disease** | **Celiac Disease** | **Esophageal Cancer** | **Hepatitis C** | **Crohn’s Disease** |
| --- | --- | --- | --- | --- | --- |
| P01023  Q03518  P29460  P48357  Q01113  O15533  P28062  P42702  P08253  P30685 | P31946  P14672  Q15796  Q92793  P06241  P63104  P62158  P84022  Q9Y4K3  P04637 | P01562  P29460  Q9HBE5  Q14213  P23743  P01589  P31785  P19397  P14784  P29353 | P00167  P00387  Q9UBK8  P11142  P14672  P08107  Q9BZE4  Q9UL45  P04637  Q92541 | P15813  P61769  Q9BXS5  P04114  P02786  P29016  P30456  P17693  P30511  P42229 | P11831  Q15599  O14745  Q5T2W1  Q86UT5  P20333  P01374  Q93038  Q9Y5U5  P08138 |
| **Breast Cancer** | **Asthma** | **Alzheimer** | **Ulcerative Colitis** | **Endometriosis** | **Lymphoma** |
| P00167  P00387  Q9UBE0  P16435  Q9UBK8  Q03135  P29460  P48357  P08047  P00451 | Q01113  Q13224  P24394  P29460  P48357  P07477  P42702  P02751  P27694  P08887 | P00740  P02647  P02652  Q9UBK8  P04070  P00742  P02768  P00734  P13500  P31946 | P01023  P11831  Q14626  P43364  P19438  Q08334  O95999  P20333  P29460  Q15599 | O14786  P49765  P17948  Q13275  P49763  P22105  P29279  P35052  P09486  P04004 | P3194  P14672  Q15796  Q92793  P06241  P63104  P62158  P84022  Q9Y4K3  O15198 |
| **Osteoarthritis** | **Epilepsy** | **Atherosclerosis** | **Pancreatitis** | **Cirrhosis** | **Myocardial Infarction** |
| Q16270  P12643  Q99985  P01584  Q99584  Q9NPH3  P02751  P21810  P09486  P07996 | P31946  P14672  Q15796  Q92793  P06241  P63104  P62158  P84022  Q9Y4K3  P04637 | P00519  P42684  P00734  P04114  P00740  Q07954  Q02156  P31946  P00747  P02652 | O60603  O60602  Q15399  Q99836  P01903  Q9BXR5  Q9Y2C9  P58753  P08571  Q9NYK1 | P17936  P24593  P22692  P08833  P18065  P24592  P19438  P20333  Q16270  P01374 | P00734  P00740  P04070  P00742  P02647  P02760  P01008  P05155  Q04756  P04004 |
| **Tuberculosis** |  |  |  |  |  |
| P01023  O95425  P54852  P16333  P62736  P00519  P62993  Q08334  P42702  P48357 |  |  |  |  |  |
